# Data-Driven Quantum Simulation of Artificial Quantum Materials with Rydberg Atoms

**DOI:** 10.3390/ma19091758

**Published:** 2026-04-25

**Authors:** Minhyuk Kim

**Affiliations:** Department of Physics, Korea University, Seoul 02841, Republic of Korea; minhyukkim@korea.ac.kr

**Keywords:** Rydberg atoms, quantum simulation, artificial quantum materials, data-driven methods, machine learning, combinatorial optimization, neural networks

## Abstract

Programmable quantum simulators based on Rydberg atom arrays provide a versatile platform for data-driven quantum simulation of strongly correlated systems, combinatorial optimization problems, and artificial quantum materials. In this review, we present a unified perspective on how materials-inspired effective Hamiltonians can be engineered and probed in Rydberg arrays, highlighting representative phenomena such as quantum phase transitions, frustrated spin-liquid–like states, symmetry-protected topological phases, and nonequilibrium dynamics. We further discuss recent progress in machine learning-based approaches, including phase identification from experimental snapshots, neural network quantum states, Hamiltonian learning, and quantum reservoir computing. A central theme is the emergence of closed-loop classical–quantum hybrid workflows, in which quantum simulation, measurement, and classical inference are integrated through iterative feedback. These developments position Rydberg atom arrays not only as programmable simulators but also as data-driven platforms for the scalable exploration, characterization, and design of complex quantum materials.

## 1. Introduction

Understanding and designing strongly correlated quantum materials remains a central challenge in condensed-matter physics [1,2,3,4]. Emergent phenomena such as magnetic frustration [5,6], topological order [7,8,9], quantum criticality [10,11,12], and nonequilibrium collective dynamics [13,14,15] arise from complex many-body interactions that generate highly structured energy landscapes. In practice, the microscopic complexity of real solids is often distilled into effective lattice Hamiltonians that capture the relevant degrees of freedom and symmetries [16,17,18,19,20,21,22,23]. Yet, determining phase diagrams, ground-state structures, and dynamical response of these interacting models is frequently computationally intractable, particularly in regimes dominated by quantum fluctuations and long-range correlations.

Programmable quantum simulators offer a complementary route to this challenge by enabling direct physical realization of model Hamiltonians under controlled and tunable conditions [24,25]. Among existing platforms, Rydberg atom arrays provide a distinctive architecture combining strong and controllable interactions, flexible geometry, and single-site resolution [26,27,28,29]. Such capabilities allow systematic exploration of parameter regimes that are difficult to access in natural materials, including frustrated lattices, constrained Hilbert spaces, and driven nonequilibrium dynamics [30]. In this sense, Rydberg arrays do not reproduce specific compounds, but rather realize synthetic quantum materials in which effective spin Hamiltonians can be engineered and probed with high precision. A natural question is how such programmable Rydberg platforms can be used to simulate and explore quantum materials, bridging theoretical models and experimentally realizable systems.

From a materials perspective, the preparation of low-energy states in programmable arrays can be viewed as controlled navigation across complex many-body energy landscapes. Adiabatic evolution [31,32], variational protocols [33], and hybrid optimization schemes provide experimentally accessible pathways toward ground states while simultaneously probing spectral gaps, critical scaling, and dynamical constraints that govern quantum phase transitions. Although some of these techniques originated in the context of combinatorial optimization, they share a common formal structure with ground-state engineering in correlated matter: both involve traversing interacting Hamiltonians whose spectral properties determine accessible phases and excitations.

The rapid growth in system size and measurement resolution of Rydberg platforms generates detailed many-body datasets, including site-resolved snapshots and multi-point correlation functions. These measurements enable systematic comparison between theoretical models and experimental realizations, supporting identification of phases, inference of effective parameters, and validation of microscopic assumptions. Data-driven approaches—such as neural network representations, phase classification algorithms, and Hamiltonian learning techniques—further extend this capability by extracting structure from experimental data and refining effective descriptions [34,35]. Rather than serving solely as standalone simulators, programmable arrays can therefore be embedded within iterative classical–quantum workflows in which experimental observations inform both model selection and control strategies [36].

**Figure 1:**
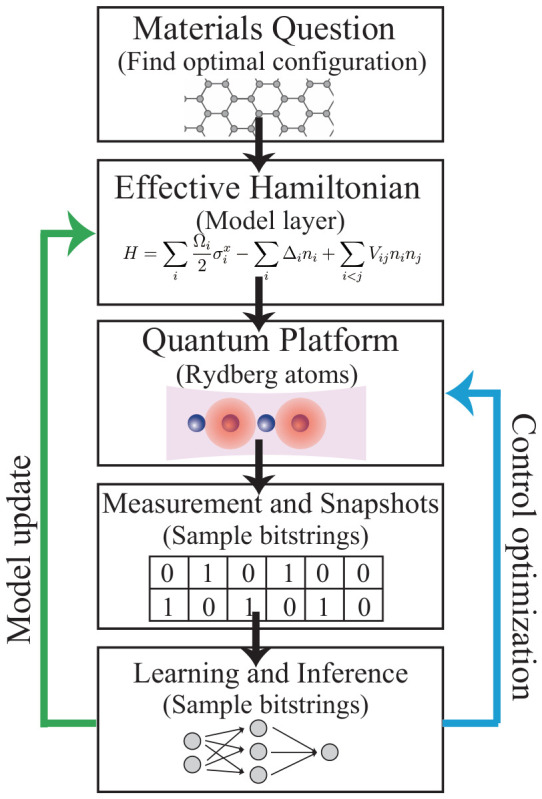
Schematic overview of a hybrid quantum–classical workflow for materials-inspired quantum simulation. A materials problem is mapped onto an effective Hamiltonian and implemented on a programmable Rydberg atom array. Measurements produce site-resolved bitstring snapshots, which are analyzed using learning and inference methods. The results are fed back to update the model and optimize control parameters, forming a closed-loop workflow.

These interconnected elements—problem formulation, Hamiltonian encoding, quantum realization, measurement, and data-driven inference—are summarized schematically in Figure 1. As illustrated, a materials question is first mapped onto an effective Hamiltonian that captures the relevant interactions and constraints. This model is then implemented on a programmable Rydberg platform, where measurements produce bitstring-resolved snapshots of the many-body state. Learning and inference procedures analyze these data to extract structure, identify optimal configurations, and guide subsequent updates. Importantly, this process forms a dual feedback loop: experimental outcomes inform both refinement of the effective Hamiltonian (model update) and optimization of experimental control parameters (control optimization), enabling iterative improvement of both the theoretical description and its physical realization.

In this paper, we review how programmable Rydberg atom arrays can be used as a unified platform for exploring artificial quantum materials, combinatorial optimization, and data-driven quantum simulation. We present a coherent framework that integrates Hamiltonian engineering, many-body state preparation, measurement, and machine learning-based analysis, emphasizing the role of closed-loop classical–quantum hybrid workflows in enabling adaptive exploration of complex quantum systems. In this perspective, the closed-loop classical–quantum workflow introduced in Figure 1 serves as a central organizing principle of this review. Accordingly, each component—Hamiltonian encoding, quantum evolution, measurement, and data-driven inference—is discussed in terms of its role within this iterative feedback framework, highlighting how programmable Rydberg platforms enable adaptive exploration of complex quantum systems. Importantly, many of the approaches discussed in this review are closely connected to experimentally realized Rydberg platforms, providing a bridge between theoretical modeling and practical implementation.

This paper is organized as follows. In Section 2, we introduce the physical principles underlying programmable Rydberg atom arrays, including effective Hamiltonian engineering and experimental implementation. In Section 3, we discuss how these systems can be used for ground-state engineering and combinatorial optimization, with a focus on mappings to classical energy landscapes. Section 4 reviews representative many-body phenomena realized in Rydberg platforms, including quantum phase transitions, frustrated spin-liquid–like states, symmetry-protected topological phases, and nonequilibrium dynamics. In Section 5, we present data-driven approaches to programmable quantum matter, highlighting machine learning techniques for phase identification, variational modeling, Hamiltonian learning, and quantum reservoir computing. Finally, we conclude with a perspective on closed-loop classical–quantum hybrid workflows and future directions for programmable quantum simulators.

## 2. Rydberg Atom Platform

Neutral-atom platforms based on optical tweezers enable deterministic trapping and manipulation of individual atoms [37]. Each atom can be initialized, coherently driven, and measured at the single-site level using precisely controlled laser fields. In particular, state-dependent fluorescence imaging allows projective measurement of individual atomic states, providing direct access to site-resolved configurations of many-body systems.

**Figure 2:**
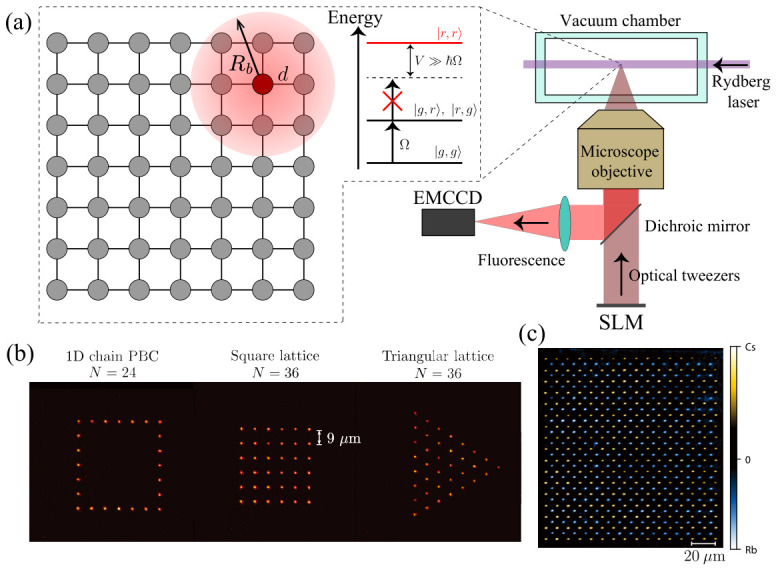
Experimental realization of Rydberg atom arrays for programmable quantum simulation. (**a**) Schematic of a neutral-atom platform implementing Rydberg blockade and an Ising-type spin Hamiltonian, including optical tweezers, Rydberg excitation lasers, and fluorescence imaging. (**b**) Examples of programmable Rydberg atom arrays with different geometries, including a one-dimensional chain with periodic boundary conditions (PBC), a square lattice, and a triangular lattice. Adapted from Ref. [47] under a CC-BY 4.0 license. (**c**) Experimental image of a two-dimensional dual-species Rydberg array composed of rubidium and caesium atoms. Adapted from Ref. [48] under a CC-BY 4.0 license.

Neutral atoms excited to high-lying Rydberg states exhibit exaggerated electromagnetic properties [38,39], including strong and long-range van der Waals [40] or dipole–dipole interactions [26,41]. Figure 2a illustrates both the physical origin of these interactions and their experimental implementation. When atoms are coherently coupled between their ground state |*g*〉 and a Rydberg state |*r*〉 by laser fields, interactions between Rydberg excitations lead to an energy shift that depends on interatomic distance. If this interaction energy exceeds the excitation linewidth, simultaneous excitation of nearby atoms becomes energetically suppressed, giving rise to the Rydberg blockade mechanism [42,43,44,45]. As a result, the excitation dynamics of the atomic ensemble become strongly correlated [46].

This mechanism naturally maps onto an effective spin-1/2 description, where |*g*〉 and |*r*〉 define the two-level basis [49]. Coherent laser driving induces transverse and longitudinal field terms, while interactions between Rydberg excitations generate distance-dependent spin couplings. The resulting effective Hamiltonian takes the form

(1) $$H=\sum_{i}\frac{\Omega_{i}}{2}\sigma_{i}^{x}-\sum_{i}\Delta_{i}n_{i}+\sum_{i<j}V_{ij}n_{i}n_{j},$$

where $\Omega_{i}$ and $\Delta_{i}$ denote the local Rabi frequency and detuning, respectively, $V_{ij}$ characterizes the interaction potential, $\sigma_{i}^{x}$ is a Pauli-*x* operator, and $n_{i}\equiv {|r\rangle }_{i}{\langle r|}_{i}$ [50,51,52]. This Hamiltonian corresponds to an extended Ising-type model with tunable transverse fields and long-range interactions, as schematically illustrated in Figure 2a.

Most current implementations employ alkali atoms such as rubidium or cesium, whose simple electronic structure and strong Rydberg interactions enable efficient trapping and coherent excitation [48,53,54,55]. More recently, alkaline-earth-like atoms including strontium and ytterbium have attracted attention due to their narrow optical transitions and long-lived metastable states, offering additional flexibility for state control and coherence [56,57,58,59]. While the choice of atomic species affects interaction strength and coherence properties, the underlying principles of Hamiltonian engineering remain broadly applicable.

### 2.1. Large-Scale and Geometry-Programmable Atomic Arrays

An important advantage of optically trapped neutral atoms is their flexible spatial arrangement [60]. Individual atoms can be assembled into defect-free arrays using optical tweezers and rearrangement protocols, enabling programmable geometries in one, two, and increasingly three dimensions [61,62,63,64,65,66]. Examples of such programmable geometries—including one-dimensional chains, square lattices, and triangular lattices—are shown in Figure 2b.

A key feature of Rydberg atom arrays is that lattice geometry itself becomes a tunable parameter. By controlling interatomic distances and connectivity, a wide range of interaction graphs can be engineered within the same experimental platform. This flexibility enables the realization of frustrated lattices and constrained Hilbert spaces, as well as the direct encoding of optimization problems into spatial configurations. It also extends to a wide range of lattice geometries, including rectangular and orthorhombic configurations, as well as more general, non-periodic arrangements.

In addition to geometric programmability, these platforms provide high-resolution measurement capabilities. After coherent evolution, atomic states are typically detected via fluorescence imaging, where atoms remaining in the ground state emit photons under resonant illumination while Rydberg-excited atoms appear dark. This mechanism enables single-site-resolved readout of many-body configurations, effectively producing bitstring snapshots of the system, as conceptually reflected in Figure 2b.

The scalability of these arrays has advanced rapidly, with experiments demonstrating coherent control of systems containing hundreds to thousands of individually addressable atoms while preserving many-body dynamics on experimentally relevant timescales [67,68]. The combination of long-range interactions, programmable geometry, and site-resolved detection establishes Rydberg atom arrays as a versatile platform for studying correlated quantum systems and implementing materials-inspired Hamiltonians. Experimental platforms have also been extended to multi-species configurations [48,55], where different atomic species can be combined within a single array to introduce additional control and interaction channels, as illustrated in Figure 2c.

### 2.2. Hamiltonian Engineering and Synthetic Quantum Matter

While Rydberg atom arrays naturally realize the transverse-field Ising Hamiltonian via van der Waals interactions, recent experiments have demonstrated that the accessible Hamiltonian space extends substantially beyond purely density–density interactions.

#### 2.2.1. Resonant Dipolar *XY* Hamiltonians

The first major extension is the implementation of long-range *XY* spin models using resonant dipole–dipole interactions between Rydberg states of opposite parity. In this regime, the effective Hamiltonian takes the form of

(2) $$H_{XY}=\sum_{i<j}U_{ij}\left(\sigma_{i}^{+}\sigma_{j}^{-}+\sigma_{i}^{-}\sigma_{j}^{+}\right),$$

where $U_{ij}=C_{3}/R_{ij}^{3}$ arises from direct dipolar exchange. The coherent propagation of a single spin excitation governed by an *XY* Hamiltonian was experimentally observed in a three-atom Rydberg chain without adjustable parameters. Furthermore, selective light shifts enable local control of resonance conditions, allowing one to tune atoms in and out of the resonant exchange regime dynamically. This capability introduces site-resolved control of exchange processes beyond static geometrical programmability [69,70].

#### 2.2.2. Heisenberg *XXZ* Models from Dipolar Interactions

By encoding spin-1/2 degrees of freedom in two Rydberg states, the full Heisenberg *XXZ* Hamiltonian can be realized. The two-atom interaction matrix directly maps onto:

(3) $$H_{XXZ}=\frac{1}{2}\sum_{i,j}J_{ij}\left(S_{i}^{x}S_{j}^{x}+S_{i}^{y}S_{j}^{y}+\delta S_{i}^{z}S_{j}^{z}\right)+\sum_{i}\Delta S_{i}^{z},$$

with $J_{ij}=2J_{ex}$ and anisotropy δ determined by the underlying dipolar matrix elements. This type of Hamiltonian was experimentally implemented in either a frozen disordered Rydberg gas [71], programmed microwave pulses [72], and external bias fields [73]. Such manipulations can readily observe the far-from-equilibrium dynamics and glassy relaxation of total magnetization.

#### 2.2.3. Toward Hubbard-Type Models

Beyond effective spin models, programmable Rydberg platforms have also been explored as analog simulators of more complex correlated-electron systems, including Hubbard-type models. In such approaches, the interplay between kinetic energy and interaction effects in fermionic systems can be recast into coupled degrees of freedom that are compatible with spin-like representations.

While Rydberg arrays do not directly simulate fermionic statistics, their native implementation of interacting spin Hamiltonians provides a flexible starting point for capturing essential features of Hubbard physics, such as interaction-driven correlations and collective behavior. This perspective opens a pathway toward studying strongly correlated electronic phenomena within programmable quantum simulators, without requiring a full digital encoding of fermionic degrees of freedom [74,75].

From a material perspective, the key feature of Rydberg atom arrays is not merely the presence of strong interactions but their controllability. The interaction strength, detuning, and driving amplitude can be dynamically tuned, enabling systematic exploration of different regions of parameter space within a single experimental setup. In this sense, Rydberg arrays realize synthetic quantum materials in which effective spin Hamiltonians are engineered rather than fixed by chemical composition or crystal structure. Such a microscopic Hamiltonian design enables controlled access to correlated phases and nonequilibrium regimes that are less accessible in natural materials.

## 3. Ground-State Engineering: From Optimization to Many-Body Energy Landscapes

The workflow introduced in Figure 1 provides a unifying perspective for understanding how programmable Rydberg platforms can be used to explore structured energy landscapes. In this section, we focus on a concrete realization of this framework through the encoding and solution of combinatorial optimization problems, and its connection to many-body physics.

### 3.1. Graph Encodings and Independent-Set Constraints

**Figure 3:**
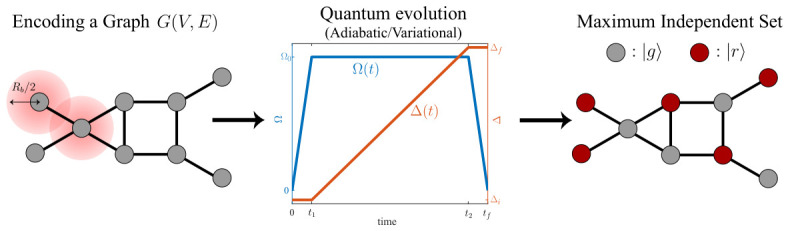
Implementation of the maximum independent set (MIS) problem using Rydberg atom arrays. A graph G(V,E) is encoded into a spatial arrangement of atoms, where edges correspond to interaction constraints mediated by the Rydberg blockade. During quantum evolution, time-dependent control parameters Ω(t) and Δ(t) drive the system toward low-energy configurations. In the final state, Rydberg excitations (red, |*r*〉) correspond to selected vertices forming an independent set, while ground-state atoms (gray, |*g*〉) denote unselected vertices.

One of the most direct applications of programmable Rydberg atom arrays is the encoding of discrete energy landscapes associated with classical combinatorial optimization problems [76]. As illustrated in Figure 3, such problems can be mapped onto interacting atomic systems in a physically transparent manner.

A graph G(V,E) is first encoded into a spatial arrangement of atoms, where each atom represents a vertex v∈V. Edges (i,j)∈E are implemented by placing atoms within the Rydberg blockade radius [77], such that simultaneous excitation of neighboring sites is energetically suppressed. In this way, the blockade mechanism directly enforces the independent-set constraint at the hardware level, restricting the accessible Hilbert space to configurations without adjacent excitations.

Under strong interactions and positive detuning Δ, the many-body ground state corresponds to the configuration that maximizes the number of Rydberg excitations while satisfying these constraints, thereby realizing a direct encoding of the maximum independent set (MIS) problem [78,79,80,81,82,83,84]. In this representation, Rydberg excitations (|*r*〉) correspond to selected vertices, while ground-state atoms (|*g*〉) represent unselected ones, as shown in Figure 3. This mapping is efficient because the interaction constraint is implemented natively by the underlying physics, eliminating the need for large penalty terms.

While early implementations focused on unit-disk graphs embedded in two-dimensional geometries, recent advances have expanded the accessible connectivity. Encoding schemes based on crossing lattices and local gadget constructions allow arbitrary graphs to be mapped onto unit-disk MIS instances with polynomial overhead [85]. Rydberg quantum wire architectures introduce auxiliary atoms that mediate effective long-range couplings, enabling the realization of non-planar graphs and high-degree vertices [80]. These developments demonstrate that programmable neutral-atom arrays are not restricted to geometrically local interactions but can engineer complex connectivity structures bridging abstract graph-based Hamiltonians with experimentally realizable atomic configurations. Beyond algorithmic interest, MIS-type formulations also arise in a broad range of materials-related and chemical optimization problems with pairwise incompatibility constraints [86,87,88,89,90].

### 3.2. Adiabatic Pathways and Spectral Gap Constraints

The encoded problem is solved through controlled quantum evolution of the interacting atomic system, as depicted in Figure 3. Time-dependent control parameters Ω(t) and Δ(t) are applied to drive the system from an initial trivial state toward a regime where interactions dominate. If the evolution is sufficiently slow compared to the inverse square of the minimum spectral gap encountered along the trajectory, the system ideally remains close to the instantaneous ground state, yielding a valid MIS solution at the end of the evolution [31,32].

From the viewpoint of correlated quantum matter, the performance of such adiabatic protocols is governed by the same spectral features that control quantum phase transitions and critical dynamics. Small spectral gaps signal enhanced correlations and critical slowing down, imposing intrinsic limits on the timescales required for ground-state preparation. Thus, adiabatic optimization and many-body critical phenomena share a common dynamical structure: both are controlled by gap closing and nonadiabatic excitations in interacting Hamiltonians.

### 3.3. Variational and Hybrid Schemes for Landscape Exploration

While adiabatic protocols rely on slow interpolation along predefined parameter trajectories, more flexible approaches treat the temporal profiles Ω(t) and Δ(t) as variational parameters to be optimized. In gate-based architectures, the quantum approximate optimization algorithm (QAOA) constructs trial states through alternating applications of problem and mixing Hamiltonians [33,91]. In analog Rydberg platforms, variational quantum adiabatic algorithms (VQAAs) similarly shape control fields and iteratively improve performance through classical feedback [92,93]. In these schemes, measurement outcomes—typically in the form of bitstring samples corresponding to atomic configurations—are used to evaluate solution quality and guide parameter updates.

This feedback-driven optimization directly reflects the closed-loop structure introduced in Figure 1. Measurement data not only encode candidate solutions but can also be used to refine control protocols and, more generally, improve effective model descriptions when discrepancies between theory and experiment arise. In this sense, solving combinatorial problems on programmable Rydberg arrays naturally fits within a broader paradigm of iterative learning and control, where quantum evolution, measurement, and classical inference are combined to explore complex many-body energy landscapes.

Beyond discrete optimization, programmable neutral-atom arrays have been employed to emulate materials-inspired energy models. In a thermodynamic sampling framework for nitrogen-doped graphene [94], DFT-derived formation energies were mapped onto a Rydberg Hamiltonian with uniform on-site and distance-dependent pair interactions. A global rescaling established correspondence between experimentally sampled distributions and those of the target material at an effective temperature, enabling grand-canonical control of composition via laser detuning. Complementarily, digital–analog hybrid approaches have embedded molecular electronic Hamiltonians into Rydberg registers and optimized pulse sequences to approximate ground-state energies with controlled accuracy [95]. These developments illustrate that variational and hybrid schemes extend programmable arrays beyond abstract graph problems toward physically motivated energy models relevant to materials and chemistry.

From the viewpoint of the closed-loop framework introduced in Figure 1, the measurement outcomes obtained from these protocols play a dual role: they not only encode candidate solutions to the optimization problem but also provide feedback for refining control parameters and improving subsequent state preparation. This connection highlights how ground-state engineering in Rydberg platforms naturally integrates into a feedback-driven, data-informed workflow.

## 4. Quantum Simulation and Control of Correlated Quantum Matter

### 4.1. Quantum Phase Transitions

Quantum phase transitions (QPTs) refer to qualitative changes in the ground state of a quantum many-body system as a non-thermal control parameter, such as the interaction strength or an external field, is tuned at zero temperature [9,96,97,98]. In contrast to classical phase transitions, which are induced by thermal fluctuations, QPTs arise from quantum fluctuations originating from competing, non-commuting terms in the Hamiltonian. A prototypical example is the transverse-field Ising model, where interactions favor symmetry-broken order while a transverse field promotes a paramagnetic state. At the critical point separating these phases, the many-body excitation gap closes in the thermodynamic limit, and correlations become long-ranged, exhibiting universal scaling behavior characterized by critical exponents that depend only on general properties such as dimensionality and symmetry [11].

**Figure 4:**
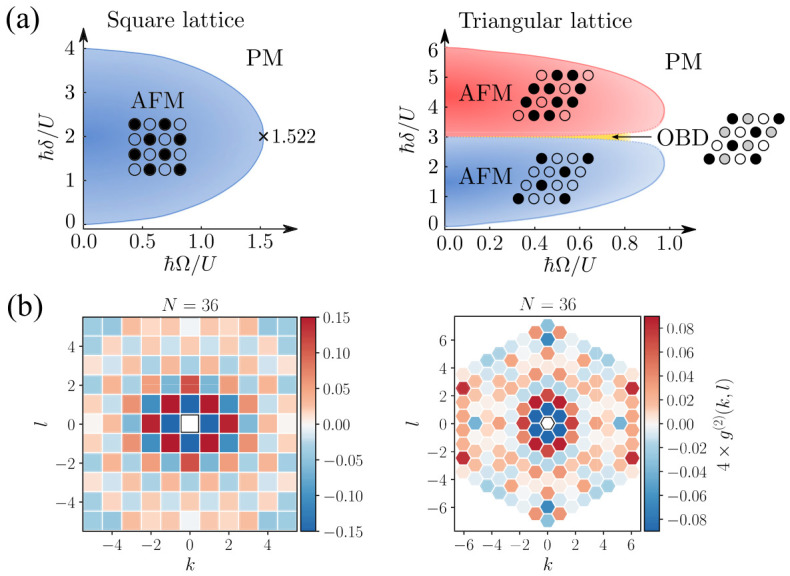
Quantum phase transitions and emergent ordering in Rydberg atom arrays. (**a**) Schematic ground-state phase diagrams of the transverse-field Ising model realized in Rydberg platforms for square (left) and triangular (right) lattice geometries, showing paramagnetic (PM) and antiferromagnetic (AFM) phases, as well as additional phases such as order-by-disorder (OBD) in the triangular case. (**b**) Experimentally measured connected correlation functions $g^{\left(2\right)}\left(k,l\right)$ for two-dimensional arrays with N=36 atoms, demonstrating short-range antiferromagnetic order in square and triangular geometries. Reprinted from Ref. [47] under a CC-BY 4.0 license.

Programmable Rydberg atom arrays provide a controlled realization of such Ising-type quantum phase transitions [99,100,101,102,103]. As summarized in Figure 4a, the competition between coherent driving and interactions gives rise to a paramagnetic (PM) phase and antiferromagnetically ordered (AFM) phases, with the precise phase structure depending on lattice geometry. In square lattices with nearest-neighbor blockade, tuning the detuning across resonance drives a transition from a paramagnetic phase into a checkerboard-ordered antiferromagnetic phase associated with spontaneous $Z_{2}$ symmetry breaking [104].

The emergence of this ordered phase can be directly observed through spatial correlations measured at the single-site level. As shown in Figure 4b, connected density–density correlations reveal the formation of short-range antiferromagnetic order, exhibiting a characteristic staggered pattern that reflects the underlying lattice geometry. Because individual atomic states can be detected with single-site resolution, observables such as correlation functions and structure factors can be extracted directly from experimental snapshots, providing microscopic access to ordering phenomena. The growth of correlations as system parameters approach the phase boundary reflects the increasing correlation length associated with the closing of the many-body excitation gap. In sufficiently large two-dimensional arrays, analyses of these correlations have yielded critical exponents consistent with the (2 + 1)-dimensional quantum Ising universality class [101,102].

The accessible phase structure becomes richer when lattice geometry or interaction range is modified. In non-bipartite geometries such as the triangular lattice, geometric frustration prevents simultaneous minimization of all interaction energies, leading to additional phases beyond simple antiferromagnetic order. As illustrated in Figure 4a, this includes density-wave phases at fractional fillings and regimes associated with order-by-disorder mechanisms [6,102,105]. Correspondingly, the measured correlation patterns deviate from simple checkerboard order and instead reflect the interplay between frustration and quantum fluctuations.

In addition to equilibrium signatures, these systems also enable exploration of dynamical phenomena when control parameters are varied in time. In particular, sweeping across a quantum phase transition at a finite rate can generate nonadiabatic excitations governed by the quantum Kibble–Zurek mechanism (QKZM) [97,106]. In this framework, the correlation length after a sweep follows a scaling relation $\xi ∼s^{-\mu }$, where the exponent μ depends on the universality class. Experiments in Rydberg systems have observed behavior consistent with these predictions, including scaling of correlation lengths and collapse of rescaled correlation functions [100,107]. Although the present discussion focuses primarily on equilibrium ordering, such dynamical scaling provides an additional perspective on how correlations develop near critical points.

Extending interactions beyond nearest neighbors further enriches the phase diagram, giving rise to competing orders such as stripe and star configurations with enlarged unit cells. In these regimes, the system reflects a balance between minimizing interaction energy and maximizing coherence induced by the transverse field. The resulting transitions can deviate from conventional Ising behavior and, in certain cases, fall into different universality classes, such as chiral clock models. Measurements of correlation growth in these regimes reveal power-law scaling behavior consistent with the corresponding universality classes within experimentally accessible parameter ranges. Together, these results demonstrate that programmable Rydberg arrays provide a versatile platform for exploring quantum phase transitions, frustrated ordering, and correlation dynamics in strongly interacting systems.

### 4.2. Quantum Spin Liquids

While the phases discussed above can be characterized by spontaneous symmetry breaking and associated local order parameters, quantum spin liquids (QSLs) represent a qualitatively different class of quantum states [9,108]. A spin liquid does not exhibit conventional long-range magnetic order even at zero temperature, despite strong interactions between spins. Instead, it is characterized by highly entangled ground states without a local order parameter, often associated with topological order defined through nonlocal properties of the many-body wavefunction. Frustrated lattice geometries provide a natural route to such states, as competing interaction constraints suppress simple ordered patterns and favor coherent superpositions of many nearly degenerate configurations [109,110].

**Figure 5:**
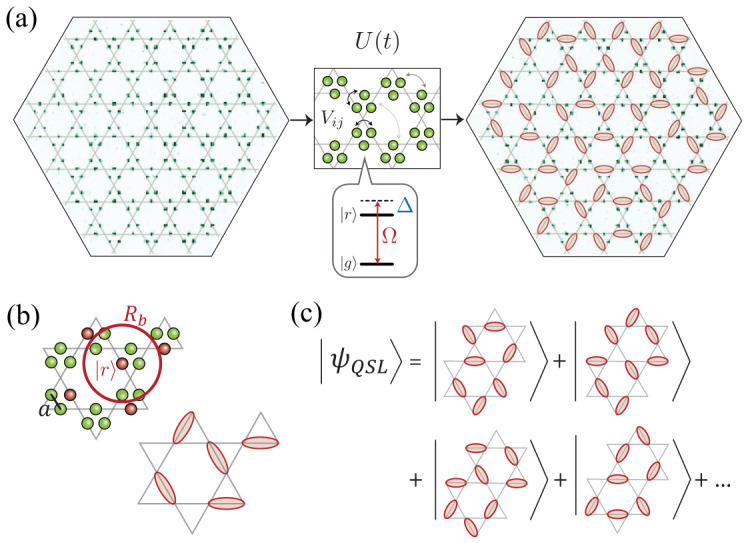
Realization of a quantum spin liquid (QSL)-like state in Rydberg atom arrays via a dimer model on a kagome geometry. (**a**) Atoms are arranged on the links of a kagome lattice and evolve under a time-dependent Rydberg Hamiltonian U(t), forming configurations consistent with dimer coverings in the strongly interacting regime. (**b**) The Rydberg blockade enforces a local constraint of at most one excitation per vertex, mapping the system onto an effective quantum dimer model. (**c**) Schematic representation of the QSL-like state as a superposition of dimer configurations. Reprinted from [111] with permission from AAAS.

Programmable Rydberg atom arrays provide a direct route to realizing such constrained and frustrated systems. As illustrated in Figure 5a, atoms can be arranged on the links of a kagome lattice and driven under a time-dependent Hamiltonian. In the regime of strong interactions, the Rydberg blockade enforces a local constraint that prevents multiple excitations within a given neighborhood. As shown in Figure 5b, this constraint effectively maps the system onto a quantum dimer model, where each vertex is occupied by at most one dimer, corresponding to a Rydberg excitation.

Within this constrained Hilbert space, the many-body state can be understood as a superposition of dimer coverings. In the idealized limit, the resulting QSL state corresponds to a coherent superposition of exponentially many such configurations, as schematically illustrated in Figure 5c. This absence of a unique ordered configuration reflects the nonlocal and highly entangled nature of the state, distinguishing it from symmetry-breaking phases.

Experimental realizations of this scenario have been demonstrated in programmable Rydberg arrays [111]. Quasi-adiabatic preparation produces states consistent with approximate dimer coverings, as indicated by measurements of Rydberg density and local constraint violations. To probe the underlying topological order, nonlocal string operators—analogous to those in the toric code—are measured. Closed-loop string operators exhibit finite signals, while corresponding open-string operators vanish over an extended parameter range, providing evidence for a spin-liquid regime.

Beyond the kagome realization, theoretical studies have explored a broader range of spin-liquid phases accessible in Rydberg platforms. In particular, mappings to emergent $Z_{2}$ gauge theories provide a framework for understanding deconfined phases and fractionalized excitations such as spinons and visons [112]. Complementary work has investigated dynamical preparation of resonating-valence-bond states using quasiadiabatic protocols [113], as well as alternative lattice geometries that may stabilize distinct spin-liquid states with more complex gauge structures [114]. These developments highlight the potential of programmable Rydberg arrays as a platform for exploring topological phases and highly entangled quantum matter.

### 4.3. Symmetry-Protected Topological Phase

Symmetry-protected topological (SPT) phases constitute a class of gapped quantum states that cannot be characterized by spontaneous symmetry breaking or local order parameters [115]. Unlike intrinsic topological phases such as quantum spin liquids, SPT states are short-range entangled and do not exhibit topological order in the sense of long-range entanglement or fractionalized excitations. Their defining feature is that the many-body ground state cannot be smoothly connected to a trivial product state without either closing the bulk excitation gap or breaking a protecting symmetry. A hallmark of SPT phases is the presence of robust edge modes under open boundary conditions, whose degeneracy is protected as long as the relevant symmetry is preserved. In one dimension, interacting bosonic SPT phases arise naturally in systems with particle-number conservation and additional discrete symmetries, extending the original Su–Schrieffer–Heeger (SSH) paradigm beyond noninteracting fermions to genuinely many-body settings.

**Figure 6:**
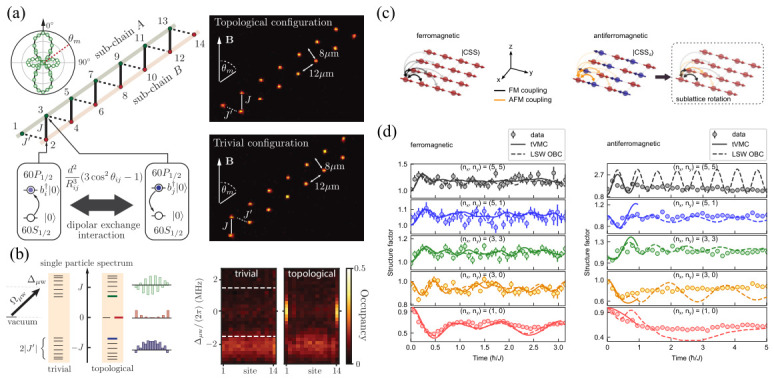
Symmetry-protected topological (SPT) phases and quench dynamics in programmable Rydberg atom arrays. (**a**) Realization of a bosonic Su–Schrieffer–Heeger (SSH)–type model using Rydberg atoms, where dipolar exchange interactions generate effective hopping between sites, with trivial and topological configurations depending on the lattice arrangement. (**b**) Single-particle and many-body spectra of the dimerized chain, showing the presence or absence of edge-localized modes. (**c**) Quench dynamics in a two-dimensional dipolar *XY* Rydberg simulator for ferromagnetic and antiferromagnetic couplings. (**d**) Time evolution of momentum-resolved structure factors following a quench for different interaction regimes. (**a**,**b**) Reprinted figures from [116]. (**c**,**d**) Reprinted figures from [118] with permission from AAAS.

Programmable Rydberg atom arrays provide a direct route to realizing such interacting topological phases. As illustrated in Figure 6a, a staggered one-dimensional chain of Rydberg atoms can implement a hard-core bosonic version of the SSH model, where dipolar exchange interactions generate effective hopping between sites. Depending on the dimerization pattern, the system realizes either a trivial phase or a topological phase. In the latter case, edge-localized modes appear together with a degenerate many-body ground state, as schematically illustrated in Figure 6b.

An experimental realization of an interacting bosonic SPT phase using Rydberg atom arrays was reported in [116]. In this work, the topological configuration exhibits a fourfold ground-state degeneracy associated with edge modes and a bulk excitation gap, whereas the trivial configuration has a unique gapped ground state. The degeneracy and edge states were probed by microwave spectroscopy, and nonlocal string order parameters were measured to distinguish the SPT phase from the trivial configuration. The robustness of the many-body ground-state degeneracy under symmetry-preserving perturbations was also demonstrated, highlighting the interacting nature of the phase beyond a single-particle description.

More recently, a disorder-induced average SPT phase was observed in a Rydberg atom array with structural disorder [117]. In this system, random displacements of optical tweezers generate fluctuating long-range dipolar couplings, breaking inversion symmetry for individual disorder realizations but restoring it on average over the ensemble. At the single-particle level, increasing disorder strength induces edge modes and a change in a generalized polarization invariant. At half-filling, the interacting system exhibits signatures consistent with a many-body average SPT phase, including statistically degenerate ground states and modified correlation functions. Microwave spectroscopy and quench dynamics measurements reveal edge-localized excitations whose behavior differs from that of bulk spins. These results extend the notion of symmetry protection to situations where the protecting symmetry holds only in an ensemble-averaged sense.

### 4.4. Non-Equilibrium Quench Dynamics

Non-equilibrium dynamics in isolated quantum many-body systems provides direct access to how correlations, excitations, and entanglement evolve following a sudden parameter change [13,119,120]. In contrast to equilibrium studies centered on static phases and ground-state properties, quench protocols reveal real-time relaxation, transport, and ergodicity-breaking phenomena. Such dynamics probe fundamental concepts including eigenstate thermalization, emergent constraints, quasiparticle propagation, and Hilbert-space structure [121,122,123]. Programmable Rydberg atom arrays offer a particularly versatile platform for these studies, enabling precise preparation of initial states, rapid quenches of Hamiltonian parameters, and site-resolved measurements of correlation functions in systems with tunable geometry and interaction range.

As illustrated in Figure 6c, different initial states can lead to qualitatively distinct dynamical evolution under otherwise similar Hamiltonians. In a two-dimensional dipolar *XY* Rydberg simulator, quench spectroscopy has been implemented by monitoring the time-dependent structure factor following a global quench [118]. From momentum-resolved oscillations of correlation functions, the dispersion relation of elementary excitations can be extracted. As shown in Figure 6d, ferromagnetic dipolar interactions produce coherent oscillations consistent with long-lived spin-wave excitations, whereas antiferromagnetic interactions lead to a more strongly damped response associated with frustration and nonlinear decay processes. In this way, quench dynamics serves not only as a probe of relaxation, but also as a spectroscopic tool for characterizing collective excitations.

Early experiments also investigated how Rydberg systems relax toward steady states after a sudden quench. In particular, relaxation dynamics in a one-dimensional Ising-like Rydberg chain were described by a master equation constructed from experimentally measured transition rates [124]. Despite the absence of an external bath, occupation probabilities obeyed a detailed-balance relation within the low-energy manifold defined by the blockade constraint, and the dynamics could be interpreted as diffusion on a graph of many-body configurations. These results provided a concrete example of how classical relaxation behavior can emerge from coherent quantum evolution in constrained Hilbert spaces.

In a complementary direction, an extremely anisotropic Heisenberg magnet realized via off-resonant Rydberg dressing has revealed further nonequilibrium phenomena [125]. In that regime, quench dynamics showed tightly and loosely bound magnon pairs, frozen motion associated with Hilbert-space fragmentation, and strongly correlated spin transport. Such experiments highlight that quench protocols can access not only elementary excitation spectra but also interaction-induced many-body effects beyond simple quasiparticle pictures.

Finally, nonequilibrium protocols enable not only probing but also control of many-body dynamics. A periodic modulation of the detuning can stabilize coherent revivals associated with quantum many-body scars, generating robust subharmonic responses reminiscent of discrete time-crystalline order [126]. Properly tuned driving parameters significantly extended the lifetime of non-ergodic oscillations and effectively engineered protected trajectories in Hilbert space. Together, these results demonstrate that programmable Rydberg arrays provide a versatile platform for exploring quantum phase transitions, frustrated ordering, and correlation dynamics in strongly interacting systems. From the perspective of the closed-loop framework introduced in Figure 1, these observables also provide essential inputs for data-driven inference and model refinement, linking many-body physics to feedback-based optimization.

## 5. Data-Driven Approaches to Programmable Quantum Matter

The programmability of Rydberg arrays not only enables the exploration of correlated phases and nonequilibrium dynamics but also generates large volumes of structured many-body data. As system sizes increase and experimental control becomes more sophisticated, extracting physical insight from these high-dimensional datasets becomes increasingly challenging. These developments naturally motivate data-driven approaches that leverage modern machine learning tools for the analysis, design, and control of programmable quantum matter.

### 5.1. Quantum Phase Identification from Experimental Snapshots

Projective measurements in programmable Rydberg atom arrays generate large ensembles of snapshot configurations, each representing a single realization of the many-body state. These data provide direct access to spatial fluctuations and correlations that are not fully captured by averaged observables. As a result, phase information is often encoded in high-dimensional statistical patterns—such as correlation functions, higher-order connected correlators, and the occurrence of local motifs—rather than in simple order parameters [127].

**Figure 7:**
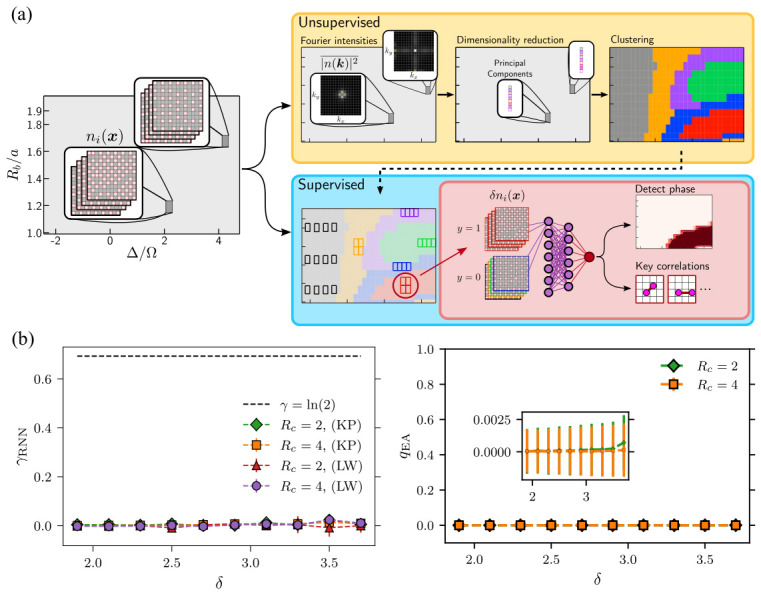
Data-driven analysis of many-body quantum states from experimental snapshots using machine learning. (**a**) Hybrid unsupervised–supervised learning pipeline applied to Rydberg atom array data, where snapshot configurations are transformed into Fourier-space features for clustering and phase identification, followed by supervised refinement using correlation-based neural networks. Reprinted from Ref. [128] under a CC-BY 4.0 license. (**b**) Neural network quantum state (NNQS) modeling of Rydberg systems using autoregressive architectures, enabling variational estimation of physical observables. Reprinted from Ref. [129] under a CC-BY 4.0 license.

Figure 7a illustrates a representative data-driven workflow for extracting such structure from experimental snapshots. In a first unsupervised stage, configurations are transformed into Fourier-space features and analyzed using dimensionality-reduction and clustering techniques, producing a coarse phase diagram. This step identifies candidate regions in parameter space without relying on prior knowledge of order parameters. In a second supervised stage, real-space fluctuation maps are used to train correlation-based neural networks, which refine phase boundaries and extract physically interpretable spatial features. Notably, this workflow is directly applicable to experimentally obtained snapshot data from programmable Rydberg platforms.

This strategy has been experimentally implemented in programmable Rydberg atom arrays [128]. The hybrid-CCNN framework combines unsupervised clustering with supervised learning to identify both known and previously unresolved phases, including edge-ordered and nematic-like structures. Importantly, the use of real-space correlator networks enables robust phase identification in finite-size systems with open boundaries, where conventional Fourier-based diagnostics become ambiguous. At the same time, interpretation of learned features requires care, as extracted correlations may reflect both intrinsic ground-state properties and effects of nonadiabatic state preparation. Nevertheless, snapshot-based machine learning provides a systematic and scalable approach to analyzing large datasets generated by programmable quantum simulators.

### 5.2. Neural Network Quantum States and Variational Modeling

Beyond classification of experimental data, machine learning can also be used to construct variational representations of many-body quantum states. Neural network quantum states (NNQSs) parameterize the wave function as $\Psi_{\theta }\left(\sigma \right)$, where σ denotes a configuration and θ are trainable parameters optimized through variational principles. This approach replaces explicit storage of exponentially many amplitudes with a compact, learnable representation.

Numerical studies have explored the application of neural network quantum states (NNQSs) to programmable Rydberg atom arrays [129]. As illustrated in Figure 7b, autoregressive architectures such as recurrent neural networks (RNNs) enable direct sampling from the probability distribution $|\Psi_{\theta }{\left(\sigma \right)|}^{2}$, avoiding the need for Markov-chain sampling and improving efficiency in frustrated systems. In practice, NNQsS are combined with variational Monte Carlo methods to approximate ground states and compute physical observables, including correlation functions and entanglement measures.

In kagome-lattice geometries, RNN-based wave functions have been used to evaluate correlation functions, topological entanglement entropy, and order parameters, providing a variational characterization of the many-body state. These results suggest that regimes previously associated with exotic phases may instead be described by simpler paramagnetic states, highlighting the importance of scalable and unbiased variational methods.

Complementary developments have explored hybrid digital–analog variational schemes tailored to Rydberg hardware [130], aiming to improve robustness and reduce circuit depth. Together, these approaches establish neural network-based variational modeling as a practical tool for connecting experimental data, effective Hamiltonians, and many-body wave functions in programmable quantum systems.

### 5.3. Hamiltonian Learning and Device Verification

**Figure 8:**
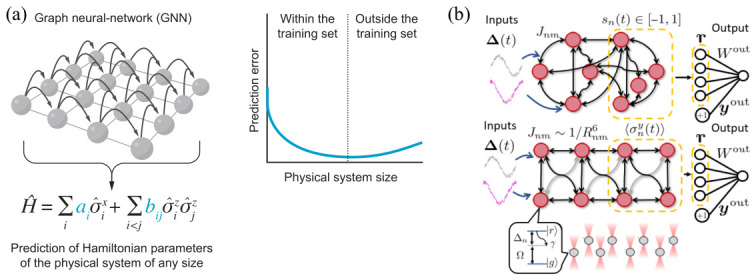
(**a**) Hamiltonian learning using graph neural networks (GNNs), where correlation functions from many-body states are encoded as graph-structured data for inference of effective Hamiltonian parameters. Reprinted from Ref. [131] under a CC-BY 4.0 license. (**b**) Quantum reservoir computing (QRC) based on Rydberg atom arrays, where time-dependent inputs are encoded through external control fields and processed via a trained readout layer. Reprinted from Ref. [132] under a CC-BY 4.0 license.

Programmable Rydberg atom arrays provide a natural setting for Hamiltonian learning, where microscopic interaction parameters are inferred directly from experimentally accessible observables. As illustrated in Figure 8a, correlation functions extracted from many-body states can be represented as graph-structured data, with nodes encoding local observables and edges encoding two-body correlations. This representation is particularly well suited for graph neural networks (GNNs), which process relational data while preserving spatial structure.

Recent work has explored Hamiltonian learning in programmable Rydberg systems through numerical studies incorporating experimentally relevant parameters and imperfections [131]. These studies demonstrate that GNN-based approaches can accurately reconstruct effective transverse-field Ising Hamiltonians from correlation measurements, even in the presence of positional disorder. A key insight underlying this approach is the existence of a bijective correspondence between spin–spin correlation functions and interaction parameters, which ensures that, in principle, experimentally measured correlations uniquely determine the underlying Hamiltonian. This provides a firm theoretical foundation for data-driven Hamiltonian inference.

An important advantage of this framework is its scalability. By construction, GNN architectures are invariant to system size and can be trained on small, classically tractable arrays while generalizing to larger systems, as reflected in Figure 8a. This enables practical deployment in experimental settings, where full classical simulation is not feasible. In this sense, Hamiltonian learning becomes an inverse problem: rather than predicting observables from a known model, one reconstructs the model itself from measurement data, providing a pathway toward device calibration, verification, and feedback-based control of programmable quantum simulators.

Complementary approaches to device characterization include ancilla-assisted quantum state tomography and machine learning-based reconstruction of effective dynamics [133,134]. While these methods rely on different measurement strategies, they share a common goal with Hamiltonian learning: extracting microscopic information from experimentally accessible data. Together, these developments illustrate a broader shift toward data-driven characterization of quantum devices.

### 5.4. Quantum Reservoir Computing with Rydberg Atom Arrays

Beyond serving as a platform for learning physical models, Rydberg atom arrays can themselves act as computational resources. Quantum reservoir computing (QRC) leverages the intrinsic dynamics of interacting quantum systems to perform machine learning tasks [132,135]. As shown in Figure 8b, time-dependent inputs are encoded into the system through external control fields, and the resulting many-body evolution generates a high-dimensional representation of the input. A simple linear readout layer is then trained to extract the desired output.

QRC with programmable Rydberg atom arrays was first explored through numerical studies [132]. This approach extends the classical reservoir paradigm to the quantum domain, where superposition, entanglement, and nonlinear many-body dynamics enrich the representational capacity of the system [135]. In contrast to variational quantum circuits, which require optimization over many parameters, reservoir computing relies on fixed Hamiltonian dynamics and trains only a small number of readout parameters, making it well suited to noisy intermediate-scale quantum (NISQ) devices. Rydberg atom arrays can serve as a natural physical platform for such quantum reservoirs, as their native spin Hamiltonians realize interacting networks analogous to recurrent neural systems [132].

The physical properties of Rydberg systems further enhance their computational capabilities. Long-range interactions generate complex nonlinear dynamics, while programmable geometries enable flexible connectivity patterns. In addition, interaction signs can be engineered through atomic-state selection, allowing effective encoding of excitatory and inhibitory couplings analogous to biological neural networks [132]. In appropriate parameter regimes, collective effects such as blockade-induced constraints and weak ergodicity breaking can give rise to enhanced memory and nontrivial dynamical responses.

Numerical studies indicate that even relatively small Rydberg arrays can perform tasks such as time-series prediction, multitasking, and decision making without requiring deep circuit architectures [136,137]. These results highlight the potential of analog quantum dynamics as a computational substrate. At the same time, important questions remain regarding scalability, robustness to noise, and the extent to which quantum dynamics provide an advantage over classical reservoir models. Ongoing work exploring noise resilience, dissipative dynamics, and hybrid classical–quantum architectures suggests that these platforms may play an increasingly important role in data-driven quantum technologies [138,139]. In this sense, data-driven approaches complete the closed-loop framework by enabling systematic extraction of structure from experimental data and feeding this information back into both model construction and control optimization.

## 6. Perspective: Toward Closed-Loop Classical–Quantum Hybrid Workflows

Programmable Rydberg atom arrays now sit at the convergence of several traditionally distinct research directions: combinatorial optimization, correlated quantum matter, nonequilibrium dynamics, and machine learning-assisted analysis. Over the past decade, Rydberg platforms have matured into versatile programmable simulators capable of engineering tunable spin Hamiltonians and exploring emergent quantum phases at increasing system sizes [28,101]. This convergence suggests that the future of the platform may be shaped less by isolated demonstrations of quantum advantage and more by increasingly integrated classical–quantum approaches, where quantum devices operate as part of a broader computational workflow.

A natural next step is the development of closed-loop experimental workflows. In this setting, quantum simulators generate high-dimensional many-body data, which are analyzed in real time using classical machine learning models. The resulting insights—such as inferred order parameters, optimized pulse schedules, or candidate Hamiltonian parameters—are then fed back to the quantum device for subsequent iterations. This perspective directly extends the closed-loop framework introduced in Figure 1, where quantum evolution, measurement, and classical inference are integrated into an iterative feedback cycle. Reinforcement learning-based quantum control and neural network-assisted state reconstruction already illustrate elements of such hybrid strategies [140,141]. These iterative cycles transform programmable quantum simulators into adaptive scientific instruments, capable of refining control strategies and exploring phase space more efficiently than purely manual approaches. In particular, optimization-based protocols, such as those used for ground-state preparation and combinatorial problems, can naturally be embedded within such feedback loops, enabling guided navigation of complex energy landscapes.

While near-term programmable Rydberg platforms already operate successfully within the NISQ paradigm [142], several experimental bottlenecks remain. Finite coherence times constitute a primary limitation, arising from multiple sources including laser noise (intensity, frequency, and phase fluctuations), spontaneous decay from Rydberg states, and the finite temperature of the atomic ensemble. In addition, maintaining large-scale, defect-free Rydberg atom arrays over extended timescales poses significant technical challenges, particularly due to limitations in laser stability and control. These experimental constraints not only restrict accessible dynamical regimes but also directly impact data-driven approaches, as the quality and reliability of measurement data are inherently tied to the stability and fidelity of the underlying quantum platform.

Looking forward, the integration of programmable quantum matter with data-driven methodologies points toward a broader shift in how complex many-body systems are studied and engineered. As illustrated in Figure 8, learning and computation can both emerge from the intrinsic dynamics of interacting quantum systems, suggesting that quantum simulators may serve not only as platforms for realizing target Hamiltonians but also as computational resources in their own right. In this perspective, tightly integrated classical–quantum workflows provide a pragmatic route toward discovery, where analog quantum devices and classical learning algorithms jointly contribute to model building, validation, and exploration. Rydberg atom arrays exemplify this shift, functioning not only as programmable simulators but as active components in adaptive, data-driven scientific processes.

## 7. Conclusions

In this review, we have presented programmable Rydberg atom arrays as a unifying platform for exploring strongly correlated quantum matter, combinatorial optimization, and data-driven quantum simulation. We discussed how effective Hamiltonians can be engineered and realized experimentally, how many-body phases and nonequilibrium dynamics can be probed through site-resolved measurements, and how classical learning methods enable systematic analysis of high-dimensional experimental data. Concrete examples—including quantum phase transitions, spin-liquid–like states, symmetry-protected topological phases, and quench dynamics—illustrate the versatility of Rydberg platforms as programmable quantum simulators.

Beyond their role as experimental realizations of model Hamiltonians, Rydberg atom arrays increasingly function as components of hybrid classical–quantum workflows. A key contribution of this review is the bridge between traditionally separate areas, such as quantum simulation, materials-inspired modeling, and data-driven approaches, providing a unified framework with relevance to both fundamental studies and emerging applications. The integration of measurement, learning, and feedback enables adaptive exploration of complex energy landscapes and provides new avenues for Hamiltonian inference and quantum-enhanced computation. Together, these developments suggest that programmable quantum matter, combined with data-driven methodologies, offers a promising route toward scalable investigation and control of complex many-body systems.

## Data Availability

No new data were created or analyzed in this study. Data sharing is not applicable to this article.

## References

[1] Dagotto E. (1994). Correlated electrons in high-temperature superconductors. Rev. Mod. Phys..

[2] Keimer B., Moore J.E. (2017). The physics of quantum materials. Nat. Phys..

[3] Defenu N., Donner T., Macrì T., Pagano G., Ruffo S., Trombettoni A. (2023). Long-range interacting quantum systems. Rev. Mod. Phys..

[4] Tokura Y. (2022). Quantum materials at the crossroads of strong correlation and topology. Nat. Mater..

[5] Ramirez A.P. (1994). Strongly Geometrically Frustrated Magnets. Annu. Rev. Mater. Res..

[6] Moessner R., Ramirez A.P. (2006). Geometrical frustration. Phys. Today.

[7] Wen X.G. (1990). Topological orders in rigid states. Int. J. Mod. Phys. B.

[8] Levin M., Wen X.G. (2006). Detecting Topological Order in a Ground State Wave Function. Phys. Rev. Lett..

[9] Wen X.G. (2017). Colloquium: Zoo of quantum-topological phases of matter. Rev. Mod. Phys..

[10] Coleman P., Schofield A.J. (2005). Quantum criticality. Nature.

[11] Sachdev S. (2008). Quantum magnetism and criticality. Nat. Phys..

[12] Sachdev S., Keimer B. (2011). Quantum criticality. Phys. Today.

[13] Polkovnikov A., Sengupta K., Silva A., Vengalattore M. (2011). Colloquium: Nonequilibrium dynamics of closed interacting quantum systems. Rev. Mod. Phys..

[14] D’Alessio L., Kafri Y., Polkovnikov A., Rigol M. (2016). From quantum chaos and eigenstate thermalization to statistical mechanics and thermodynamics. Adv. Phys..

[15] Heyl M. (2018). Dynamical quantum phase transitions: A review. Rep. Prog. Phys..

[16] Hohenberg P., Kohn W. (1964). Inhomogeneous Electron Gas. Phys. Rev..

[17] Kohn W., Sham L.J. (1965). Self-Consistent Equations Including Exchange and Correlation Effects. Phys. Rev..

[18] Ceperley D.M., Alder B.J. (1980). Ground State of the Electron Gas by a Stochastic Method. Phys. Rev. Lett..

[19] Jones R.O. (2015). Density functional theory: Its origins, rise to prominence, and future. Rev. Mod. Phys..

[20] Metropolis N., Rosenbluth A.W., Rosenbluth M.N., Teller A.H., Teller E. (1953). Equation of State Calculations by Fast Computing Machines. J. Chem. Phys..

[21] McMillan W.L. (1965). Ground State of Liquid He^4^. Phys. Rev..

[22] Hastings W.K. (1970). Monte Carlo sampling methods using Markov chains and their applications. Biometrika.

[23] Anderson J.B. (1975). A Random-Walk Simulation of the Schrödinger Equation: $H_{3}^{+}$. J. Chem. Phys..

[24] Georgescu I.M., Ashhab S., Nori F. (2014). Quantum simulation. Rev. Mod. Phys..

[25] Daley A.J., Bloch I., Kokail C., Flannigan S., Pearson N., Troyer M., Zoller P. (2022). Practical quantum advantage in quantum simulation. Nature.

[26] Saffman M., Walker T.G., Mølmer K. (2010). Quantum information with Rydberg atoms. Rev. Mod. Phys..

[27] Saffman M. (2016). Quantum computing with atomic qubits and Rydberg interactions: Progress and challenges. J. Phys. B At. Mol. Opt. Phys..

[28] Browaeys A., Lahaye T. (2020). Many-body physics with individually controlled Rydberg atoms. Nat. Phys..

[29] Morgado M., Whitlock S. (2021). Quantum simulation and computing with Rydberg-interacting qubits. AVS Quantum Sci..

[30] Weimer H., Müller M., Lesanovsky I., Zoller P., Büchler H.P. (2010). A Rydberg quantum simulator. Nat. Phys..

[31] Farhi E., Goldstone J., Gutmann S., Sipser M. (2000). Quantum Computation by Adiabatic Evolution. arXiv.

[32] Albash T., Lidar D.A. (2018). Adiabatic quantum computation. Rev. Mod. Phys..

[33] Farhi E., Goldstone J., Gutmann S. (2014). A Quantum Approximate Optimization Algorithm. arXiv.

[34] Henry L.P., Thabet S., Dalyac C., Henriet L. (2021). Quantum evolution kernel: Machine learning on graphs with programmable arrays of qubits. Phys. Rev. A.

[35] Albrecht B., Dalyac C., Leclerc L., Ortiz-Gutiérrez L., Thabet S., D’Arcangelo M., Cline J.R.K., Elfving V.E., Lassablière L., Silvério H. (2023). Quantum feature maps for graph machine learning on a neutral atom quantum processor. Phys. Rev. A.

[36] Zheng M., Zeng J., Yang W., Chang P.J., Lu Q., Yan B., Zhang H., Wang M., Wei S., Long G.L. (2025). Quantum-classical hybrid algorithm for solving the learning-with-errors problem on NISQ devices. Commun. Phys..

[37] Schlosser N., Reymond G., Protsenko I., Grangier P. (2001). Sub-poissonian loading of single atoms in a microscopic dipole trap. Nature.

[38] Gallagher T.F. (1994). Rydberg Atoms.

[39] Lim J., Lee H.G., Ahn J. (2013). Review of cold Rydberg atoms and their applications. J. Korean Phys. Soc..

[40] Béguin L., Vernier A., Chicireanu R., Lahaye T., Browaeys A. (2013). Direct Measurement of the van der Waals Interaction between Two Rydberg Atoms. Phys. Rev. Lett..

[41] Browaeys A., Barredo D., Lahaye T. (2016). Experimental investigations of dipole–dipole interactions between a few Rydberg atoms. J. Phys. B At. Mol. Opt. Phys..

[42] Jaksch D., Cirac J.I., Zoller P., Rolston S.L., Côté R., Lukin M.D. (2000). Fast Quantum Gates for Neutral Atoms. Phys. Rev. Lett..

[43] Lukin M.D., Fleischhauer M., Cote R., Duan L.M., Jaksch D., Cirac J.I., Zoller P. (2001). Dipole Blockade and Quantum Information Processing in Mesoscopic Atomic Ensembles. Phys. Rev. Lett..

[44] Urban E., Johnson T.A., Henage T., Isenhower L., Yavuz D.D., Walker T.G., Saffman M. (2009). Observation of Rydberg blockade between two atoms. Nat. Phys..

[45] Gaëtan A., Miroshnychenko Y., Wilk T., Chotia A., Viteau M., Comparat D., Pillet P., Browaeys A., Grangier P. (2009). Observation of collective excitation of two individual atoms in the Rydberg blockade regime. Nat. Phys..

[46] Wilk T., Gaëtan A., Evellin C., Wolters J., Miroshnychenko Y., Grangier P., Browaeys A. (2010). Entanglement of Two Individual Neutral Atoms Using Rydberg Blockade. Phys. Rev. Lett..

[47] Lienhard V., de Léséleuc S., Barredo D., Lahaye T., Browaeys A., Schuler M., Henry L.P., Läuchli A.M. (2018). Observing the Space- and Time-Dependent Growth of Correlations in Dynamically Tuned Synthetic Ising Models with Antiferromagnetic Interactions. Phys. Rev. X.

[48] Singh K., Anand S., Pocklington A., Kemp J.T., Bernien H. (2022). Dual-Element, Two-Dimensional Atom Array with Continuous-Mode Operation. Phys. Rev. X.

[49] de Léséleuc S., Weber S., Lienhard V., Barredo D., Büchler H.P., Lahaye T., Browaeys A. (2018). Accurate Mapping of Multilevel Rydberg Atoms on Interacting Spin-$1/2$ Particles for the Quantum Simulation of Ising Models. Phys. Rev. Lett..

[50] Labuhn H., Barredo D., Ravets S., de Léséleuc S., Macrì T., Lahaye T., Browaeys A. (2016). Tunable two-dimensional arrays of single Rydberg atoms for realizing quantum Ising models. Nature.

[51] Schauss P. (2018). Quantum simulation of transverse Ising models with Rydberg atoms. Quantum Sci. Technol..

[52] Kim M., Song Y., Kim J., Ahn J. (2020). Quantum Ising Hamiltonian Programming in Trio, Quartet, and Sextet Qubit Systems. PRX Quantum.

[53] Graham T.M., Song Y., Scott J., Poole C., Phuttitarn L., Jooya K., Eichler P., Jiang X., Marra A., Grinkemeyer B. (2022). Multi-qubit entanglement and algorithms on a neutral-atom quantum computer. Nature.

[54] Singh K., Bradley C.E., Anand S., Ramesh V., White R., Bernien H. (2023). Mid-circuit correction of correlated phase errors using an array of spectator qubits. Science.

[55] Anand S., Bradley C.E., White R., Ramesh V., Singh K., Bernien H. (2024). A dual-species Rydberg array. Nat. Phys..

[56] Cooper A., Covey J.P., Madjarov I.S., Porsev S.G., Safronova M.S., Endres M. (2018). Alkaline-Earth Atoms in Optical Tweezers. Phys. Rev. X.

[57] Madjarov I.S., Covey J.P., Shaw A.L., Choi J., Kale A., Cooper A., Pichler H., Schkolnik V., Williams J.R., Endres M. (2020). High-fidelity entanglement and detection of alkaline-earth Rydberg atoms. Nat. Phys..

[58] Jenkins A., Lis J.W., Senoo A., McGrew W.F., Kaufman A.M. (2022). Ytterbium Nuclear-Spin Qubits in an Optical Tweezer Array. Phys. Rev. X.

[59] Peper M., Li Y., Knapp D.Y., Bileska M., Ma S., Liu G., Peng P., Zhang B., Horvath S.P., Burgers A.P. (2025). Spectroscopy and Modeling of ^171^*Yb* Rydberg States for High-Fidelity Two-Qubit Gates. Phys. Rev. X.

[60] Nogrette F., Labuhn H., Ravets S., Barredo D., Béguin L., Vernier A., Lahaye T., Browaeys A. (2014). Single-Atom Trapping in Holographic 2D Arrays of Microtraps with Arbitrary Geometries. Phys. Rev. X.

[61] Endres M., Bernien H., Keesling A., Levine H., Anschuetz E.R., Krajenbrink A., Senko C., Vuletic V., Greiner M., Lukin M.D. (2016). Atom-by-atom assembly of defect-free one-dimensional cold atom arrays. Science.

[62] Barredo D., de Léséleuc S., Lienhard V., Lahaye T., Browaeys A. (2016). An atom-by-atom assembler of defect-free arbitrary two-dimensional atomic arrays. Science.

[63] Kim H., Kim M., Lee W., Ahn J. (2019). Gerchberg-Saxton algorithm for fast and efficient atom rearrangement in optical tweezer traps. Opt. Express.

[64] Barredo D., Lienhard V., de Léséleuc S., Lahaye T., Browaeys A. (2018). Synthetic three-dimensional atomic structures assembled atom by atom. Nature.

[65] Lin R., Zhong H.S., Li Y., Zhao Z.R., Zheng L.T., Hu T.R., Wu H.M., Wu Z., Ma W.J., Gao Y. (2025). AI-Enabled Parallel Assembly of Thousands of Defect-Free Neutral Atom Arrays. Phys. Rev. Lett..

[66] Schymik K.N., Lienhard V., Barredo D., Scholl P., Williams H., Browaeys A., Lahaye T. (2020). Enhanced atom-by-atom assembly of arbitrary tweezer arrays. Phys. Rev. A.

[67] Manetsch H.J., Nomura G., Bataille E., Lv X., Leung K.H., Endres M. (2025). A tweezer array with 6,100 highly coherent atomic qubits. Nature.

[68] Chiu N.C., Trapp E.C., Guo J., Abobeih M.H., Stewart L.M., Hollerith S., Stroganov P.L., Kalinowski M., Geim A.A., Evered S.J. (2025). Continuous operation of a coherent 3000-qubit system. Nature.

[69] Barredo D., Labuhn H., Ravets S., Lahaye T., Browaeys A., Adams C.S. (2015). Coherent Excitation Transfer in a Spin Chain of Three Rydberg Atoms. Phys. Rev. Lett..

[70] de Léséleuc S., Barredo D., Lienhard V., Browaeys A., Lahaye T. (2017). Optical Control of the Resonant Dipole-Dipole Interaction between Rydberg Atoms. Phys. Rev. Lett..

[71] Signoles A., Franz T., Ferracini Alves R., Gärttner M., Whitlock S., Zürn G., Weidemüller M. (2021). Glassy Dynamics in a Disordered Heisenberg Quantum Spin System. Phys. Rev. X.

[72] Scholl P., Williams H.J., Bornet G., Wallner F., Barredo D., Henriet L., Signoles A., Hainaut C., Franz T., Geier S. (2022). Microwave Engineering of Programmable *XXZ* Hamiltonians in Arrays of Rydberg Atoms. PRX Quantum.

[73] Kunimi M., Tomita T. (2025). Proposal for realizing Heisenberg-type quantum-spin models in Rydberg-atom quantum simulators. Phys. Rev. A.

[74] Michel A., Henriet L., Domain C., Browaeys A., Ayral T. (2024). Hubbard physics with Rydberg atoms: Using a quantum spin simulator to simulate strong fermionic correlations. Phys. Rev. B.

[75] Julià-Farré S., Michel A., Domain C., Mikael J., Lafoucriere J.C., Vovrosh J., Chahlaoui A., Claveau D., Villaret G., Hond J.D. (2025). Hybrid quantum-classical analog simulation of two-dimensional Fermi-Hubbard models with neutral atoms. arXiv.

[76] Lucas A. (2014). Ising formulations of many NP problems. Front. Phys..

[77] Kim M., Ahn J., Song Y., Moon J., Jeong H. (2023). Quantum computing with Rydberg atom graphs. J. Korean Phys. Soc..

[78] Pichler H., Wang S.T., Zhou L., Choi S., Lukin M.D. (2018). Quantum Optimization for Maximum Independent Set Using Rydberg Atom Arrays. arXiv.

[79] Ebadi S., Keesling A., Cain M., Wang T.T., Levine H., Bluvstein D., Semeghini G., Omran A., Liu J.G., Samajdar R. (2022). Quantum optimization of maximum independent set using Rydberg atom arrays. Science.

[80] Kim M., Kim K., Hwang J., Moon E.G., Ahn J. (2022). Rydberg quantum wires for maximum independent set problems. Nat. Phys..

[81] Byun A., Kim M., Ahn J. (2022). Finding the Maximum Independent Sets of Platonic Graphs Using Rydberg Atoms. PRX Quantum.

[82] Dalyac C., Henry L.P., Kim M., Ahn J., Henriet L. (2023). Exploring the impact of graph locality for the resolution of the maximum-independent-set problem with neutral atom devices. Phys. Rev. A.

[83] Kim K., Kim M., Park J., Byun A., Ahn J. (2024). Quantum computing dataset of maximum independent set problem on king lattice of over hundred Rydberg atoms. Sci. Data.

[84] de Oliveira A.G., Diamond-Hitchcock E., Walker D.M., Wells-Pestell M.T., Pelegrí G., Picken C.J., Malcolm G.P.A., Daley A.J., Bass J., Pritchard J.D. (2025). Demonstration of Weighted-Graph Optimization on a Rydberg-Atom Array Using Local Light Shifts. PRX Quantum.

[85] Nguyen M.T., Liu J.G., Wurtz J., Lukin M.D., Wang S.T., Pichler H. (2023). Quantum Optimization with Arbitrary Connectivity Using Rydberg Atom Arrays. PRX Quantum.

[86] Jeong S., Kim M., Hhan M., Park J., Ahn J. (2023). Quantum programming of the satisfiability problem with Rydberg atom graphs. Phys. Rev. Res..

[87] Park J., Jeong S., Kim M., Kim K., Byun A., Vignoli L., Henry L.P., Henriet L., Ahn J. (2024). Rydberg-atom experiment for the integer factorization problem. Phys. Rev. Res..

[88] Byun A., Jung J., Kim K., Kim M., Jeong S., Jeong H., Ahn J. (2024). Rydberg-Atom Graphs for Quadratic Unconstrained Binary Optimization Problems. Adv. Quantum Technol..

[89] Leclerc L., Dalyac C., Bendotti P., Griset R., Mikael J., Henriet L. (2025). Implementing transferable annealing protocols for combinatorial optimization on neutral-atom quantum processors: A case study on smart charging of electric vehicles. Phys. Rev. A.

[90] D’Arcangelo M., Henry L.P., Henriet L., Loco D., Gouraud N., Angebault S., Sueiro J., Forêt J., Monmarché P., Piquemal J.P. (2024). Leveraging analog quantum computing with neutral atoms for solvent configuration prediction in drug discovery. Phys. Rev. Res..

[91] Zhou L., Wang S.T., Choi S., Pichler H., Lukin M.D. (2020). Quantum Approximate Optimization Algorithm: Performance, Mechanism, and Implementation on Near-Term Devices. Phys. Rev. X.

[92] Schiffer B.F., Tura J., Cirac J.I. (2022). Adiabatic Spectroscopy and a Variational Quantum Adiabatic Algorithm. PRX Quantum.

[93] Bombieri L., Zeng Z., Tricarico R., Lin R., Notarnicola S., Cain M., Lukin M.D., Pichler H. (2025). Quantum Adiabatic Optimization with Rydberg Arrays: Localization Phenomena and Encoding Strategies. PRX Quantum.

[94] Camino B., Lin M., Buckeridge J., Woodley S.M. (2025). Thermodynamic sampling of materials using neutral-atom quantum computers. arXiv.

[95] Michel A., Grijalva S., Henriet L., Domain C., Browaeys A. (2023). Blueprint for a digital-analog variational quantum eigensolver using Rydberg atom arrays. Phys. Rev. A.

[96] Sachdev S. (2011). Quantum Phase Transitions.

[97] Zurek W.H., Dorner U., Zoller P. (2005). Dynamics of a Quantum Phase Transition. Phys. Rev. Lett..

[98] Vojta M. (2003). Quantum phase transitions. Rep. Prog. Phys..

[99] Bernien H., Schwartz S., Keesling A., Levine H., Omran A., Pichler H., Choi S., Zibrov A.S., Endres M., Greiner M. (2017). Probing many-body dynamics on a 51-atom quantum simulator. Nature.

[100] Keesling A., Omran A., Levine H., Bernien H., Pichler H., Choi S., Samajdar R., Schwartz S., Silvi P., Sachdev S. (2019). Quantum Kibble–Zurek mechanism and critical dynamics on a programmable Rydberg simulator. Nature.

[101] Ebadi S., Wang T.T., Levine H., Keesling A., Semeghini G., Omran A., Bluvstein D., Samajdar R., Pichler H., Ho W.W. (2021). Quantum phases of matter on a 256-atom programmable quantum simulator. Nature.

[102] Scholl P., Schuler M., Williams H.J., Eberharter A.A., Barredo D., Schymik K.N., Lienhard V., Henry L.P., Lang T.C., Lahaye T. (2021). Quantum simulation of 2D antiferromagnets with hundreds of Rydberg atoms. Nature.

[103] Samajdar R., Ho W.W., Pichler H., Lukin M.D., Sachdev S. (2021). Quantum phases of Rydberg atoms on a kagome lattice. Proc. Natl. Acad. Sci. USA.

[104] Samajdar R., Ho W.W., Pichler H., Lukin M.D., Sachdev S. (2020). Complex Density Wave Orders and Quantum Phase Transitions in a Model of Square-Lattice Rydberg Atom Arrays. Phys. Rev. Lett..

[105] Kim K., Chang M.S., Korenblit S., Islam R., Edwards E.E., Freericks J.K., Lin G.D., Duan L.M., Monroe C. (2010). Quantum simulation of frustrated Ising spins with trapped ions. Nature.

[106] Dziarmaga J. (2005). Dynamics of a Quantum Phase Transition: Exact Solution of the Quantum Ising Model. Phys. Rev. Lett..

[107] Zhang T., Wang H., Zhang W., Wang Y., Du A., Li Z., Wu Y., Li C., Hu J., Zhai H. (2025). Observation of Near-Critical Kibble-Zurek Scaling in Rydberg Atom Arrays. Phys. Rev. Lett..

[108] Zhou Y., Kanoda K., Ng T.K. (2017). Quantum spin liquid states. Rev. Mod. Phys..

[109] Han T.H., Helton J.S., Chu S., Nocera D.G., Rodriguez-Rivera J.A., Broholm C., Lee Y.S. (2012). Fractionalized excitations in the spin-liquid state of a kagome-lattice antiferromagnet. Nature.

[110] Fu M., Imai T., Han T.H., Lee Y.S. (2015). Evidence for a gapped spin-liquid ground state in a kagome Heisenberg antiferromagnet. Science.

[111] Semeghini G., Levine H., Keesling A., Ebadi S., Wang T.T., Bluvstein D., Verresen R., Pichler H., Kalinowski M., Samajdar R. (2021). Probing topological spin liquids on a programmable quantum simulator. Science.

[112] Samajdar R., Joshi D.G., Teng Y., Sachdev S. (2023). Emergent *Z*_2_ Gauge Theories and Topological Excitations in Rydberg Atom Arrays. Phys. Rev. Lett..

[113] Giudici G., Lukin M.D., Pichler H. (2022). Dynamical Preparation of Quantum Spin Liquids in Rydberg Atom Arrays. Phys. Rev. Lett..

[114] Kornjača M., Samajdar R., Macrì T., Gemelke N., Wang S.T., Liu F. (2023). Trimer quantum spin liquid in a honeycomb array of Rydberg atoms. Commun. Phys..

[115] Senthil T. (2015). Symmetry-Protected Topological Phases of Quantum Matter. Annu. Rev. Condens. Matter Phys..

[116] de Léséleuc S., Lienhard V., Scholl P., Barredo D., Weber S., Lang N., Büchler H.P., Lahaye T., Browaeys A. (2019). Observation of a symmetry-protected topological phase of interacting bosons with Rydberg atoms. Science.

[117] Yue Z., Mao Y.F., Liang X., Hua Z.X., Ge P., Chao Y.X., Li K., Jia C., Tey M.K., Xu Y. (2025). Observation of average topological phase in disordered Rydberg atom array. arXiv.

[118] Chen C., Emperauger G., Bornet G., Caleca F., Gély B., Bintz M., Chatterjee S., Liu V., Barredo D., Yao N.Y. (2025). Spectroscopy of elementary excitations from quench dynamics in a dipolar XY Rydberg simulator. Science.

[119] Lieb E.H., Robinson D.W. (1972). The finite group velocity of quantum spin systems. Commun. Math. Phys..

[120] Mitra A. (2018). Quantum Quench Dynamics. Annu. Rev. Condens. Matter Phys..

[121] Deutsch J.M. (1991). Quantum statistical mechanics in a closed system. Phys. Rev. A.

[122] Srednicki M. (1994). Chaos and quantum thermalization. Phys. Rev. E.

[123] Rigol M., Dunjko V., Olshanii M. (2008). Thermalization and its mechanism for generic isolated quantum systems. Nature.

[124] Kim H., Park Y., Kim K., Sim H.S., Ahn J. (2018). Detailed Balance of Thermalization Dynamics in Rydberg-Atom Quantum Simulators. Phys. Rev. Lett..

[125] Kim K., Yang F., Mølmer K., Ahn J. (2024). Realization of an Extremely Anisotropic Heisenberg Magnet in Rydberg Atom Arrays. Phys. Rev. X.

[126] Bluvstein D., Omran A., Levine H., Keesling A., Semeghini G., Ebadi S., Wang T.T., Michailidis A.A., Maskara N., Ho W.W. (2021). Controlling quantum many-body dynamics in driven Rydberg atom arrays. Science.

[127] Carrasquilla J. (2020). Machine learning for quantum matter. Adv. Phys. X.

[128] Miles C., Samajdar R., Ebadi S., Wang T.T., Pichler H., Sachdev S., Lukin M.D., Greiner M., Weinberger K.Q., Kim E.A. (2023). Machine learning discovery of new phases in programmable quantum simulator snapshots. Phys. Rev. Res..

[129] Hibat-Allah M., Merali E., Torlai G., Melko R.G., Carrasquilla J. (2025). Recurrent neural network wave functions for Rydberg atom arrays on kagome lattice. Commun. Phys..

[130] Lu J.Z., Jiao L., Wolinski K., Kornjača M., Hu H.Y., Cantu S., Liu F., Yelin S.F., Wang S.T. (2024). Digital–analog quantum learning on Rydberg atom arrays. Quantum Sci. Technol..

[131] Simard O., Dawid A., Tindall J., Ferrero M., Sengupta A.M., Georges A. (2025). Learning Interactions between Rydberg Atoms. PRX Quantum.

[132] Bravo R.A., Najafi K., Gao X., Yelin S.F. (2022). Quantum Reservoir Computing Using Arrays of Rydberg Atoms. PRX Quantum.

[133] Kim K., Ahn J. (2023). Quantum Tomography of Rydberg Atom Graphs by Configurable Ancillas. PRX Quantum.

[134] Mukherjee K., Schachenmayer J., Whitlock S., Wüster S. (2025). Quantum network tomography of small Rydberg arrays by machine learning. Phys. Rev. A.

[135] Mujal P., Martínez-Peña R., Nokkala J., García-Beni J., Giorgi G.L., Soriano M.C., Zambrini R. (2021). Opportunities in Quantum Reservoir Computing and Extreme Learning Machines. Adv. Quantum Technol..

[136] Llodrà G., Mujal P., Zambrini R., Giorgi G.L. (2024). Quantum reservoir computing in atomic lattices. arXiv.

[137] Zhu C., Ehlers P.J., Nurdin H.I., Soh D. (2025). Minimalistic and scalable quantum reservoir computing enhanced with feedback. Npj Quantum Inf..

[138] Sannia A., Martínez-Peña R., Soriano M.C., Giorgi G.L., Zambrini R. (2024). Dissipation as a resource for Quantum Reservoir Computing. Quantum.

[139] Tariq S., Talha M., Chatzinotas S., Shin H. (2025). Towards Quantum Enhanced Adversarial Robustness with Rydberg Reservoir Learning. arXiv.

[140] Bukov M., Day A.G.R., Sels D., Weinberg P., Polkovnikov A., Mehta P. (2018). Reinforcement Learning in Different Phases of Quantum Control. Phys. Rev. X.

[141] Torlai G., Timar B., van Nieuwenburg E.P.L., Levine H., Omran A., Keesling A., Bernien H., Greiner M., Vuletić V., Lukin M.D. (2019). Integrating Neural Networks with a Quantum Simulator for State Reconstruction. Phys. Rev. Lett..

[142] Preskill J. (2018). Quantum Computing in the NISQ era and beyond. Quantum.

